# Supplementary material for: Self-assembly of sodium dodecylsulfate and dodecyltrimethylammonium bromide mixed surfactants with dyes in aqueous mixtures
Source: R Soc Open Sci. 2019 Mar 27;6(3):181979. doi: 10.1098/rsos.181979 (PMC6458362; doi:10.1098/rsos.181979)
Supplement: Self-assembly of SDS and DTAB mixed surfactants with dyes in aqueous mixtures [file rsos181979supp1.docx]

Self-assembly of SDS and DTAB mixed surfactants with dyes in aqueous mixtures

K. M. Sachin^1^, Sameer A. Karpe^1^, Man Singh^1^, Ajaya Bhattarai^1, 2^*

^1^School of Chemical Sciences, Central University of Gujarat, Gandhinagar, India

^2^Department of Chemistry, M.M. A. M. Campus, Tribhuvan University, Biratnagar, Nepal

Author for correspondence: *Ajaya Bhattarai ([bkajaya@yahoo.com](mailto:bkajaya@yahoo.com))

**(a)**

**(b)**

**Figure S1.** Plot of Specific Conductivity versus concentration of SDS-rich (a) and DTAB-rich (b) solution at 293.15(○), 298.15(□) and 303.15 K (Δ).

**Figure S2.** Plot of $\Delta S_{m}^{o}$versus $\Delta H_{m}^{o}$ for DTAB-rich and SDS-rich micellar solutions.

**Figure S3**$\boldsymbol{.} \Delta H_{m}^{o}$versus Temperature curve for calculation of heat capacity of micellization of SDS-rich (○) and DTAB-rich (Δ).

**Figure S4.** UV-visible data of MB/ DTAB-SDS/water system: lines are suitable fitting curves obtained in NLREG procedure.

**Figure S5.** UV-visible data of MO/ DTAB-SDS /water system: lines are suitable fitting curves obtained in NLREG procedure.
